# Insights into nervous system repair from the fruit fly

**DOI:** 10.1042/NS20210051

**Published:** 2022-04-13

**Authors:** David Coupe, Torsten Bossing

**Affiliations:** Peninsula Medical School, University of Plymouth, John Bull Building, 16 Research Way, Plymouth PL6 8BU, U.K.

**Keywords:** axon regrowth, axonal injury, cell proliferation, CNS damaga, glial injury

## Abstract

Millions of people experience injury to the central nervous system (CNS) each year, many of whom are left permanently disabled, providing a challenging hurdle for the field of regenerative medicine. Repair of damage in the CNS occurs through a concerted effort of phagocytosis of debris, cell proliferation and differentiation to produce new neurons and glia, distal axon/dendrite degeneration, proximal axon/dendrite regeneration and axon re-enwrapment. In humans, regeneration is observed within the peripheral nervous system, while in the CNS injured axons exhibit limited ability to regenerate. This has also been described for the fruit fly *Drosophila*. Powerful genetic tools available in *Drosophila* have allowed the response to CNS insults to be probed and novel regulators with mammalian orthologs identified. The conservation of many regenerative pathways, despite considerable evolutionary separation, stresses that these signals are principal regulators and may serve as potential therapeutic targets. Here, we highlight the role of *Drosophila* CNS injury models in providing key insight into regenerative processes by exploring the underlying pathways that control glial and neuronal activation in response to insult, and their contribution to damage repair in the CNS.

## Introduction

Injury to the CNS extensively impacts the lives of patients, their relatives and the community at large. Each year, 69 million people worldwide suffer a traumatic brain injury (TBI) [1]. Brain injuries are the biggest contributor to death and disability of all trauma induced injuries. Survivors of TBI often undergo a long convalescence and/or will suffer life changing consequences. Thus, research into the mechanisms by which damage occurs and may be repaired remains critical. Due to the similarities in the development and function of the CNS in bilateral organisms [2], the study of model organisms that can partially or completely regenerate their CNS is an interesting research area which yields potential translational insight into mechanisms of human neural regeneration.

Damage to the CNS affects three different components: axons/dendrites, neuronal cell bodies and glia cells. The observation that peripheral axons can regenerate to a certain extent whereas central axons cannot, indicates that different mechanisms regulate axonal regeneration in the CNS [3]. The lack of axonal regeneration is never more apparent than in spinal cord repair after traumatic injuries. The fight against paralysis after spinal cord fracture is an intense and dynamic research field and excellent reviews are available [4–7]. In this report, we will mainly focus on injury and repair after brain damage.

The neural regenerative abilities of mammals are limited, with studies of adult neurogenesis focusing on the classical niches for neural stem cells (NSCs) located in the subventricular zone (SVZ) of the lateral ventricle and the subgranular zone (SGZ) of the dentate gyrus. Additional smaller niches have been described for the circumventricular organs, the lining of the third ventricle which forms part of the hypothalamus, the walls of the fourth ventricle and the central canal of the spinal cord [8–10].

In response to traumatic brain injury all stem cell niches in the rodent brain can increase proliferation [11]. Many studies have focused on the SVZ and central canal ependymal cells, showing stem cell proliferation increases and neuroblasts sometimes migrate to the site of injury and differentiate [10]. Increased local proliferation and differentiation, into glia and neurons, is also observed in the SGZ of the dentate gyrus, but little cell migration has been observed [9]. A contribution to functional recovery by the newly born neurons is very limited. Synapses between SVZ-derived neuroblasts and neighboring cells have been identified and some evidence of functional recovery was reported [12]. Removal of SVZ-derived cells following stroke recovery also hampers recovery of motor function [13]. However, the majority of newly generated neurons fail to integrate into extant circuits and die [14–16].

### Similarities between human and *Drosophila* CNS repair

While vertebrates with regenerative capacities such as zebrafish, salamander and axolotl provide valuable insight [9], the use of model organisms with limited regenerative abilities is also informative. Both adult humans and *Drosophila* fruit flies repair but not fully regenerate CNS lesions [17]. In flies and in mammals, axonal breakage leads to retraction and degeneration of the distal stump. Inhibitory extrinsic factors and lack of intrinsic growth capacity prevent axonal regrowth [18]. Initial clearance of apoptotic cells and fragmented axons require microglia in mammals, while several glial types including microglia-like cells (MiC) [19] perform this role in *Drosophila* [20]. The clearance leads to lesion expansion as both glia and neurons die, the subsequent shrinkage correlates with repair and regenerative processes. CNS lesion triggers proliferation of astrocytes and NG2 glia in mammals [21]. NG2 glia, astrocytes, microglia, monocytes/ macrophages, epithelial cells, fibroblasts and oligodendrocyte precursors agglomerate at the injury site to form a glial scar, presenting an obstacle for axonal regrowth [5]. In *Drosophila*, astrocyte-like glia (ALG) proliferate but no glial scar is formed [22]. Damage triggers glia proliferation producing a limited number of ensheathing glia (EG) to re-enwrap axons [17]. Traumatic head injuries in humans and flies activate the innate immune response by triggering Toll and TNF signaling. In both species activation of the innate response is linked to impaired recovery [23,24]. In humans and flies injury to the CNS leads to permanent disability [9,17,22,25]. These similarities combined with the powerful genetics and well characterized development of *Drosophila*, allow fruit fly CNS injury models to provide insight into mechanisms of human CNS repair and regeneration.

## *Drosophila* CNS injury models

Several methods have been developed to study injury and repair in the fruit fly CNS *in vivo* and *ex vivo*. Ablation of midline cells in the embryo damages the developing CNS and results in additional divisions replacing damaged cells [26]. Larval damage to the ventral nerve cord (VNC; functionally equivalent to the spinal cord), by either *ex vivo* stabbing [27] or *in vivo* crush [28] induce a stereotypical glial regenerative response (GRR). In adults, injury models comprise axotomy of the small lateral neurons ventral (sLNv) in cultured brains [29], crush injury of the metathoracic neuromere (MtN) segment of the adult VNC [30] and stab injury to the optic lobe [31].

Regeneration in peripheral nervous system (PNS) and CNS neurons differ [3] but insights from the study of *Drosophila* PNS injury have also provided valuable insights into mammalian regeneration of the CNS and PNS. Models used include larval segmental nerve crush [32], wing transection [33] and peripheral neuron laser axotomy [34]. Additionally, the injury response has been explored using axotomy of sensory neurons that cross between the CNS and PNS, namely class IV dendritic arborisation (C4da) neurons [35] and olfactory receptor neurons (ORNs) [36,37]. Following axotomy within the PNS, C4da neurons are able to regenerate. Yet, within the CNS the axons of the same neurons are unable to regrow [35]. These models allow the response and regenerative potential of the same axons in both, CNS and PNS, to be studied, aiding the identification of factors that promote or inhibit regeneration to be identified.

## Neuronal response to injury

The regeneration of severed axons is limited by extrinsic and intrinsic factors. In mammals and flies, extrinsic environmental differences between the CNS and PNS lead to differences in axon regeneration [35]. In mammals, the interaction with axonal Nogo-Receptors (NgRs) and glycoproteins on the surrounding myelin sheath such as Nogo, myelin-associated glycoprotein (MAG) and oligodendrocyte myelin glycoprotein (OMgp) inhibits axonal regrowth [38]. In fruit flies, axons are not enwrapped in myelin. Also, in cold-blooded animals, including *Drosophila*, a glial scar is not formed. Due to differences in the extrinsic environment and limited success in targeting external regeneration modulators [39,40], much of the work using *Drosophila* has focused on understanding and manipulating intrinsic pathways, with particular emphasis on the process of Wallerian degeneration (WD).

### Response of axons to injury: Wallerian degeneration (WD)

Transected axons retract and begin to swell before fragmenting. In the proximal axon, filopodia-like outgrowths form but stall after a matter of days and do not cross the lesion. In the distal axon, an active degeneration process, WD, is initiated [29,41,42].

Central to the understanding of WD was the chance discovery of the *slow Wallerian degeneration* (*Wld^S^*) mouse [43], in which axon stumps distal to an injury survive and function for weeks rather than only a couple of days, following axotomy. The protein resulting from the *Wld^S^* mutation is a fusion of the 70 N-terminal residues of the E4 ubiquitin ligase Ube4b (N70), a linker domain, and the complete NAD^+^ biosynthetic enzyme nicotinamide-nucleotide adenylyltransferase 1 (Nmnat1). *Wld^S^* has also been found to provide protection against axon degeneration when expressed in *Drosophila* [36] and zebrafish [44], but the process of WD in *Caenorhabditis elegans* appears to be unaffected by Wld^S^ [45]. The role of Ube4b-N70 is unclear as there is no apparent difference in ubiquitination between wild-type (wt) and *Wld^S^* mice [46]. Consequently, much of the focus of research has been on the role of NMNAT genes.

In contrast to humans and mice which have three NMNAT genes (Table 1), *Drosophila* only has one Nmnat gene which is most similar to mammalian NMNAT1 and NMNAT3. The function of *Drosophila Nmnat (dNmnat)* is still under investigation. *dNmnat* has been observed to slow WD after axotomy of the peripheral axon of wing neurons [33] and olfactory receptor neurons (ORNs) [36], as well as protect against neuronal degeneration in response to traumatic brain injury [47].

**Table 1:** Genes regulating CNS repair in *Drosophila* and their mammalian orthologues

Location of repair after CNS damage	Drosophila gene	Mammalian ortholog	DIOPT score	Function	Function in mammalian CNS damage/repair
**Neuronalr epair response**					
**Axon**	Axundead (axed)	–	–	–	
	F-box synaptic protein (Fsn)	Fbxo45	13/15	E3 ubiquitin ligase	
	Highwire (hiw)	Myc-binding protein 2 (Mycbp2)	14/15	E3 ubiquitin ligase	[48]
	Nmnat	NMNAT1/3	13/15	NAD synthesis	[43]
	Sterile alpha/Armadillo/Toll-interleukin receptor homology domain protein (sarm)	Sarm1	10/15	NAD+ depletion	[55]
	Skp1a	SKP1	14/15	E3 ubiquitin ligase	[95,96]
	TER 94	VCP	15/15	Ubiquitin dependant chaperone	[41,97,98]
**Neuron**	Akt kinase (Akt)	AKT	14/15	Cell signaling	[99]
	Archease	ZBTB8OS	14/15	Enzymatic turnover of rtcb	[68,100]
	Down syndrome cell adhesion molecule 1 (Dscam1)	DSCAM	13/15	Cell adhesion	[100]
	Fat facets (faf)	USP9X	14/15	Ubiquitinyl hydrolase	[101]
	futsch	MAP1A/ MAP1B	8/15	Microtubule binding	[70,102]
	Grindelwald (Grnd)	-	-	TNF receptor	
	Histone deacytelase 6 (hdac6)	HDAC6	14/15	Histone deacytelase, protein misfolding response	[103,104]
	oo18 RNA-binding protein (Orb)	CPEB1	8/15	Cytoplasmic polyadenylation element (CPE) binding protein	[105]
	Phosphatase and tensin homolog (pten)	Pten	13/15	Phosphatase	[106,107]
	Rtcb Rna ligase (rtcb)	RTCB	13/15	RNA ligase	[67,68]
	Ringmaker (ringer)	TPPP	12/15	Microtubule polymerization	[108]
	RNA 3′-teminal phosphate cyclase (rtca)	RTCA	12/15	RNA cyclase	[67,68]
	Wallenda (wnd)	MAP3K13	10/15	MAP kinase kinase kinase	[109,110]
	Xbox-binding protein 1 (xbp1)	XBP1	10/15	Unfolded protein response	[111]
**Glial repair response**					
**Debris clearance**	corkscrew (csw)	PTPN11	12/15	Nonreceptor tyrosine phosphatase	[112]
	basket (bsk)	MAPK8	13/15	Ser/thr protein kinase	[113]
	ced6	GULP1	9/15	Adaptor protein	[114]
	Draper	MEGF10	12/15	Phagocytosis	[72,114]
	Flower	CACFD1	12/15	Transmembrane protein	[115]
	Jun-related antigen (jra)	JUND	11/15	Jun transcription factor	[116–118]
	Kayak (kay)	FOS	6/15	Fos-related transcription factor	[119]
	MAP kinase kinase 4 (MKK4)	MAP2K4	15/15	Mitogen-activated protein kinase kinase	[120,121]
	Matrix metalloproteinase 1 (Mmp1)	MMP14/ MMP24	9/15	Proteinase	[122]
	misshapen (msn)	TNIK	13/15	MAP kinase kinase kinase	[123,124]
	SH2 ankyrin repeat kinase (Shark)	ZAP70	7/15	Nonreceptor tyrosine kinase	[125]
	Rac1	RAC1	12/15	GTPase	[126,127]
	Rho1	RHOA	13/15	GTPase	[126,127]
	Son of sevenless (sos)	SOS1	14/15	Ras/ Rho Guanine nucleotide exchange factor	[128]
	Src orthologue at 42A (Src42A)	FRK	14/15	Nonreceptor tyrosine kinase	[129]
	TGF-β activated kinase 1 (Tak1)	MAP3K7	9/15	MAP kinase kinase kinase	[130]
	TNF receptor associated factor 4 (Traf4)	TRAF4	14/15	Adaptor protein binding TNF receptor	[131]
**Glial proliferation**	deadpan (dpn)	HES1/ HES4	7/15	Transcriptional repressor/ stemness marker	[132,133]
	dacapo (dap)	CDKN1A	3/15	Cyclin-dependent kinase inhibitor	[134]
	dorsal (dl)	RELA	9/15	Transcription factor	[135,136]
	Eiger (egr)	EDA	6/15	TNF ligand	[135]
	Ia2 protein tyrosine phosphatase (ia2)	PTPRN	11/15	Protein tyrosine phosphatase	[137]
	Insulin-like peptide 6 (Ilp6)	–	–	Neuropeptide	
	kon-tiki (kon)	CSPG4	13/15	Transmembrane protein	[138–140]
	Myc	MYC	6/15	Transcription factor	[141,142]
	Notch (N)	NOTCH1	12/15	Transmembrane signaling receptor	[132,143,144]
	prospero (pros)	PROX1	10/15	Transcription factor	[145,146]
	Wengen (wgn)	–	–	TNF receptor	
**Midline repair response**					
**Midline proliferation**	Cactus (cact)	NFKBIA	10/15	NfkappaB binding; KappaB inhibitor	[147]
	dorsal (dl)	RELA	9/15	Transcription factor	[135,136]
	Eiger (egr)	EDA	6/15	TNF ligand	[135]
	Jun-related antigen (jra)	JUND	11/15	Jun transcription factor	[116–118]
	Mitochondrial Rho (Miro)	RHOT1	14/15	Mitochondrial Rho GTPase; microtubule binding	[148,149]
	TGF-β activated kinase 1 (Tak1)	MAP3K7	9/15	MAP kinase kinase kinase	[94,121]
	IkappaB kinase ε Ik2	TBK1	13/15	TANK-binding kinase	[94,150,151]

DIOPT score from www.flybase.org.

**Figure 1:**
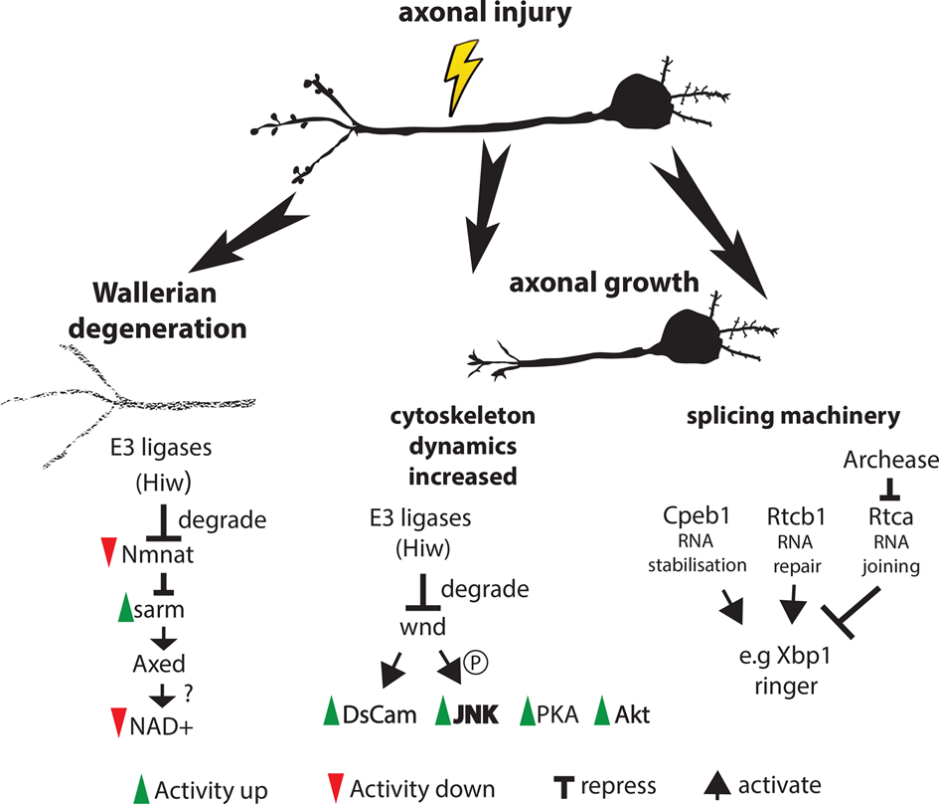
Axonal injury triggers the removal of the distal and the growth of the proximal axon Removal of the distal axonal fragment starts with the degradation of Nmnat, which is targeted by E3 ligases. Loss of Nmat releases sarm repression, resulting in activation of Axed, NAD+ Nucleosidases and NAD+ depletion and culminating in Wallerian degeneration. Sarm and Axed interact genetically. How Axed influences NAD+ Nucleosidase activity is not known. Distal axonal fragments must be removed to allow the growth of proximal axon stumps. Activation of DsCam, PKA and Akt and JNK signaling is required for axonal re-growth. Wnd promotes axonal re-growth by phosphorylation and enhancement of DsCam and JNK signaling. E3 ligases target Wnd to counteract axonal re-growth. Changes in the splicing machinery stabilise extant RNAs promoting axon extension after injury; Axed, Axundead; CPEB, cytoplasmic polyadenylation element binding; Dscam1, Down syndrome cell adhesion molecule 1; Hiw, Highwire; JNK, c-jun n-terminal kinase; PKA, Protein kinase A; Rtca, RNA 3′-terminal phosphate cyclase; Rtcb, RNA 2′,3′-cyclic phosphate and 5′-OH ligase; Ringer, microtubule stabilizer, Sarm, Sterile alpha/Armadillo/Toll-interleukin receptor homology domain protein; Wnd, Wallenda; Xbp1, X-box binding protein 1.

In mammals, the proposed mechanism by which NMNATs degradation activates distal axon degradation focuses on NMNAT2 (Figure 1). NMNAT2 is distributed by axonal trafficking. When axons are severed, WD occurs at a rate that correlates with the half-life of NMNAT2 [40,42]. In *Drosophila*, *Highwire (hiw)*, an E3 ubiquitin ligase and ortholog of the human MYC binding protein 2 (Mycbp2) (Table 1), has been identified as one of the regulators of dNmnat, promoting its degradation at the distal axon tip. Degradation of NMNAT2 performed by Mycbp2 is also observed in mice [48]. Other E3 ligases that target NMNATs are Skp1a and Fbxo45 [49]. In summary, the presence of NMNATs prevent, whereas its absence promotes axonal degradation.

Several studies using enzyme inactive NMNATs have been used to further elucidate mechanisms. When dNmnat is lost in photoreceptor neurons, development proceeds normally but results in early degeneration, a phenotype that can be rescued by the expression of enzyme-dead dNmnat, suggesting that the neuroprotective abilities of dNmnat are not conferred by its enzymatic activity [50]. An explanation for how enzyme dead dNmnat can provide neuroprotection may be presented by its role as a chaperone, which has also been demonstrated for mouse NMNAT2 [50] and human NMNAT3 [51]. In contrast, in ORNs, expression of an enzyme dead dNmnat does not prevent decay of the distal axon stump after injury. NMNAT1 expression delays axon degeneration but is not as efficient as Wld^S^. Interestingly a fusion protein of the 16 N-terminal amino acids of Wld^S^ with NMNAT1 is as efficient in prevention of axon decay as the full length Wld^S^. This functional rescue is thought to depend on binding to the ubiquitinating co-factor VCP/TER94 [52].

While NMNAT proteins demonstrate important axonal protective properties, the mechanisms by which NMNATs act in homeostasis, disease and injury are less clear and remain controversial [40,49]. A potential explanation of the tissue specific differences in the ability of NMNAT to prevent axon decay may be an interaction with neuron specific co-factors as seen in the ORNs.

In forward genetic screens to identify mutants with delayed WD within the brain after antennal ablation or severed wing sensory neurons, *Drosophila sterile alpha/Armadillo/Toll-interleukin receptor homology domain protein (sarm)* [53] and *axundead (axed)* [54] have been identified (Figure 1). Loss of *sarm* can slow ORN axon degeneration to a similar extent as *Wld^S^* expression and preserves the axonal cytoskeleton. *Sarm* has an identified ortholog in mice (Table 1), *Sarm1*, that is also required for normal WD in PNS and CNS axons [53,55]. As in *Drosophila*, loss of Sarm1 slows down axonal degradation and improves functional recovery after TBI [56]. In dorsal root ganglion (DRG) neurons Sarm1 dimers drive axonal degradation by activating depletion of NAD^+^ following axotomy [57]. Loss of axed can rescue the deleterious effects of dSarm and loss of *dNmnat*, indicating it acts as downstream of both *dSarm* and *dNmnat* [54]. *Drosophila axed* has no obvious mammalian ortholog.

One of the regulators of dNmnat degradation is *Highwire (hiw*), a E3 ubiquitin ligase. In *hiw* mutants WD takes significantly longer following crush of larval segmental nerves [32] and TBI [47]. Loss of *hiw* slows WD by slowing dNmnat degradation. From these assays, a model has emerged in which E3 ubiquitin ligases, including Hiw, are master regulators of WD, leading to the depletion of NMNATs and subsequent axonal degeneration [32].

At first glance the slowing of axon decay after injury seems advantageous. Yet, NMNAT’s rescue of the distal axon creates a significant obstacle for regrowth of the proximal axon that requires a clearance of debris [32,58]. It is thought that NMNATs may also indirectly act to inhibit PI3K-mTORC regenerative signaling [59]. An example of the biphasic role of NMNATs in response to axon injury is shown during ddaE sensory neuron axotomy, in which dNmnat degradation is required for normal WD to occur whereas its overexpression reduces the regrowth of the proximal axon stump normally observed [58]. Delayed clearance of the distal axonal stump may also explain the slowdown in regeneration of the sensory and motor neurons of the *Wld^S^* mouse [60,61].

### Injury response from the soma

In response to injury, the c-Jun N-terminal kinase (JNK) signaling pathway is up-regulated in both neurons and glia [29,30]. Increased JNK signaling in the small lateral neurons ventral (sLNv) of the adult fruit fly brain, promotes axonal regrowth, to the extent that a small proportion of severed axons can traverse the lesion. However, inactivation of JNK signaling does not significantly alter regeneration, suggesting other pathways may initiate regeneration, possibly via protein kinase A (PKA) [29] or PTEN-Akt signaling [35] (Figure 1).

Eiger (Egr), the canonical JNK-pathway ligand and tumor necrosis factor (TNF) ortholog in *Drosophila*, is required for activation of JNK signaling in many CNS injury paradigms [62]. Yet, although Grindelwald *(grnd)*, an Egr receptor, is required for activation of JNK signalling in response to adult MtN segment crush injury, Egr itself is dispensable [30]. Other JNK-pathways, independent of Egr, have also been identified, with *wallenda (wnd)*, the ortholog of Map3K13 (Table 1), being a key regulator of non-canonical JNK signalling [63].

As well as regulating NMNATs, Hiw also targets Wnd, reducing JNK signaling and dampening axon outgrowth in response to axotomy [32]. *Fat facets (Faf)*, the fly ortholog of *Usp9x I* (Table 1), antagonizes Hiw by de-ubiqitination in the development of neuromuscular junctions [64] and appears to perform the same function in response to CNS injury. Via noncanonical JNK signaling, Wnd is able to stabilize Down syndrome cell adhesion molecule 1 (Dscam1) mRNA via the 3′UTR [63]. Dscam1 isoforms have been implicated in axonal self-recognition and self-avoidance, dendritic patterning and regulate presynaptic branches, yet it is unclear how increased Dscam1 can promote axon regeneration. Co-overexpression of Faf and Dscam1 leads to stronger outgrowth of the sLNv than overexpression of either protein alone [63], suggesting a novel neuronal regenerative pathway.

Signaling via PTEN-Akt has been implicated in the differential abilities of the CNS and PNS to regenerate. The axonal terminals in the CNS of C4da sensory neurons are unable to regenerate but peripheral axonal segments of the same neuron exhibit regenerative outgrowth after damage. An increase in Akt signaling by Akt overexpression or via mutating Pten, leads to increased outgrowth following axotomy within the CNS [35], suggesting a mechanism involved in the different regenerative capabilities between the CNS and PNS. Excitingly, using optogenetic control, it has been demonstrated that Akt signaling, and Raf/MEK/ERK signaling, can be spatially and temporally activated in injured CNS and PNS axons, leading to functional recovery of thermonociception, for which C4da neurons are essential [65]. In summary, activation of a noncanonical JNK signaling in concert with Pten-Akt signaling in neurons are the main driving force for axonal regrowth. Pten-Akt signaling also modulates axonal regrowth according to the environment—CNS or PNS.

Cellular stress conditions can trigger a global shut down in translational operations but selective translation of specific transcripts is important in axonal regeneration. Transcripts with a decline of translation were identified in a combined approach comparing the transcriptome and translatome in mice and mapping the total RNA and polysome-bound RNA in a *Drosophila* screen. Identified transcripts were implicated in CNS development, cell death, transcription and RNA processing and the immune response. While the genes identified to be involved in CNS development showed reduced expression, translation of mRNAs of this category was maintained [66]. A subset of these genes is enriched in the cytoplasmic polyadenylation element (CPE) 3′UTR motif, which is bound by the *Drosophila* CPE-binding protein, Orb, and mammalian ortholog, Cpeb1 (Table 1). Indeed, overexpression of *Orb* in sLNv in *Drosophila* and *Cpeb1* in *mouse* retinal ganglion cells promotes axon regeneration [66].

In the C4da axotomy model, RNA processing has also been identified to be an important regulator of regeneration (Figure 1). Cellular stress due to injury can damage RNAs, which can be repaired by the RNA ligase, Rtcb (RNA 2′,3′-cyclic phosphate and 5′-OH ligase). Repair occurs in a two-stage process. First the 2′,3′-cyclic phosphodiester must be converted to a 3′-phosphate, a process that is antagonised by RNA 3′-terminal phosphate cyclase (Rtca). Second, the 3′-phosphate of one RNA molecule is then joined to the 5′-OH of another, enhanced by Archease [67]. Therefore, Rtca acts to inhibit axon regeneration and Archease can promote regeneration. Overexpression of *Rtca* inhibits the normal regenerative abilities of C4da neurons following axotomy within the PNS, while *Rtca* loss enhances regeneration following axotomy within the CNS. The regenerative enhancement after axotomy within the CNS is dependent on X-box binding protein 1 (Xbp1), a transcription factor that coordinates transcription in response to stress. This regulatory pathway is broadly conserved (Table 1). In rats, following peripheral sciatic nerve injury, Rtca transcription is reduced in the dorsal root ganglion (DRG) but is not reduced in response to lesion of the DRG within the spinal cord. Rtca transcriptional regulation represents a possible mechanism by which regeneration is inhibited within the CNS but permitted within the PNS [68].

A subsequent transcriptomic screen of *Rtca* mutants, identified *ringer* as upregulated in response to C4da neuron axotomy within the PNS. Ringer was identified together with the microtubule-associated protein futsch/MAP1B to stimulate regeneration. Futsch/ MAP1B promotes microtubule stability/dynamics [69], a process that is known to promote regeneration [70]. In support of a critical role of microtubule stability, regeneration is inhibited by microtubule-associated deacetylase HDAC6 which destabilises microtubules [69].

In summary, activation of a noncanonical JNK signaling in concert with Akt signaling in neurons are the main driving force for axonal regrowth. Akt signaling also modulates axonal regrowth according to the environment—CNS or PNS. Axonal injury also activates cellular stress pathways which stop *de novo* transcription but stabilizes extant RNA thereby promoting the synthesis of microtubule associated proteins which stabilize axonal growth.

## The glial regenerative response (GRR) to injury

**Figure 2:**
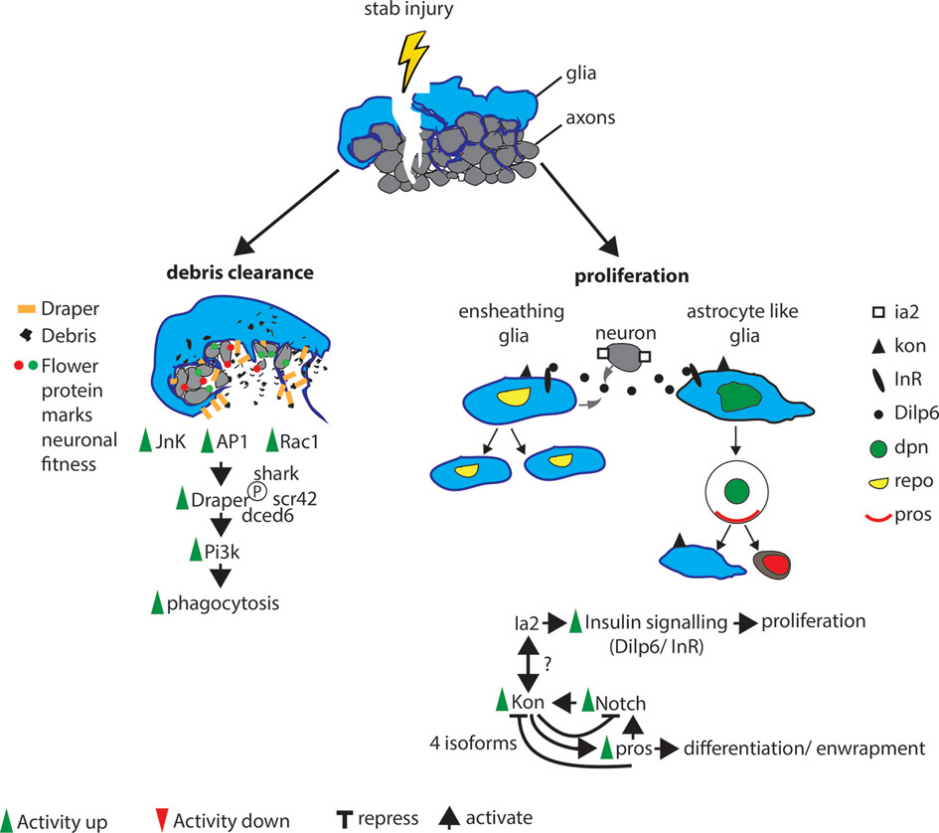
Glial injury activates debris clearance and glial proliferation Stab injury to the CNS damages glial cells and axons. Glial cells in the vicinity of the damage site up-regulate JNK signaling and thereby formation of the AP1 transcription heterodimer. In addition, Rac1/ Rho signalling activation by small GTPases results in cytoskeleton remodeling and increased Draper expression. Draper activation and debris engulfment depends on phosphorylation by Src42, Shark and the adaptor molecule Ced6. Draper activation up-regulates Pi3K signaling in adjacent undamaged neurons blocking axonal transport and hindering recovery. Damage also stimulates proliferation of glial cells. Ia-2 on neuronal membranes genetically interacts with Kon, the NG2 ortholog, on glia membranes. Activation of Ia-2 and Kon activates neuronal and glial secretion of Dilp6, which binds to InR on glia initiating insulin signaling and proliferation. In a subset of ensheathing glia cells that express the transcription factor repo, Insulin signaling drives Cyclin E, initiating division. Active Notch signaling promotes proliferation whereas nuclear incorporation of Pros terminates proliferation. In some astrocyte-like glia, insulin signaling initiates Dpn expression, a neural stem cell marker. Dpn positive cells divide to give rise to neurons and glia. The PDZ domain of Kon can be cleaved by alpha- and gamma-secretases into four different isoforms, of which two are part of a negative feedback loop repressing Notch signaling and activating Pros expression; AP1, activator protein 1; Dilp6, Drosophila insulin like peptide 6; Dpn, Deadpan; Ia-2, Islets antigen-2; InR, Insulin receptor; JNK, c-jun n-terminal kinase; Kon, Kon-tiki; NG2, Neural glia antigen 2; Pros, Prospero; Repo, Reverse polarity; Src42, Sarcoma 42

The response by glia to CNS injury is a highly stereotypical process that can be categorised into stages: clearance of debris, proliferation and differentiation [22,30,36] (Figure 2). These stages, and many of the gene networks that underlie them, are conserved in flies and mammals. *Drosophila* glia are classified differently than mammalian glia. *Drosophila* neuropile glia (NG) are either astrocyte-like glia (ALG) or ensheathing glia (EG) [71], and functionally equivalent to mammalian astrocytes, NG2 glia and oligodendrocytes, both in undamaged and damaged CNS. ALG share many properties with both mammalian astrocytes and NG2 glia, as they interact closely with synapses and express neurotransmitter transporters, such as *ebony*, a dopamine/ histamine transporter, and VGAT, a vesicular GABA transporter. Yet, they differ from astrocytes in that they to do not express one of the main mammalian astrocyte marker, the intermediate filament glial fibrillary acidic protein (GFAP) nor *Megf10* [22] that mediates the clearance of apoptotic neurons [72]. Draper, the *Drosophila* ortholog of Megf10 (Table 1), is however found on EG, where it is an essential glial engulfment receptor for the clearance of damaged axons [36]. EG enwrap axons, similar to mammalian oligodendrocytes, but do not synthesize myelin or form nodes of Ranvier. EG also express neurotransmitter re-uptake transporters, such as excitatory amino acid transporter1 *(eaat1)* [73], and *glutamine synthetase 2*, regulating glutamine synthesis [74]. These factors allow glia to regulate transmitter amount at the synaptic cleft and to modulate the neuronal response [75]. ALG and NG2 glia also share the central regulators of the GRR, Notch, Prospero (Pros) and Kon-tiki (Kon), the *Drosophila* NG2 ortholog (Table 1) [22].

### Tidying up: clearance of debris

Damage to the *Drosophila* CNS activates phagocytosis in two cell types. After crush injury to the adult ventral nerve cord, phagocytic hemocytes, which are functionally equivalent to vertebrate macrophages, enter the nerve cord. Genetic depletion of hemocytes limits functional recovery [30]. In response to insult, all glia can become phagocytic. Phagocytosis in glia cells requires the expression of the engulfment receptor, Draper [22,37,73] (Figure 2). EG enwrap the neuropile, providing a barrier between the neuropile and the cortex. After axotomy EG extend membranes into the neuropile. Since axonal debris exhibits limited diffusion from the site of the lesion only local EG respond [36]. A knockdown of Draper prevents EG membrane extensions [73]. Brain damage also affects the survival of neurons. A darwinian-like selection process has been identified to mediate the clearance and replacement of damaged neurons within the optic lobe, whereby specific isoforms of Flower, ortholog of the human calcium channel Flower domain-containing protein 1 (CACFD1) (Table 1), mark a neuron as “unfit” and as target for clearance by glial cells [76] (Figure 2).

Ligands for Draper in response to CNS injury remain unknown but likely can be found in the degradation products of neurons. This is indicated by the block of axonal degradation and thereby the production of degeneration products in *hiw* mutants. In *hiw* mutants ORN axotomy initiates Draper expression but signalling via Draper seems blocked since no morphological changes in EG glia can be observed [77]. In the optic lobe, Pretaporter, CaBP1 and phosphatidylserine have been suggested as ligands for Draper, as their suppression delays the clearance of dead neurons [78,79].

Draper expression, activation and localisation have been investigated in some detail. Insulin-like signaling, through InR, Akt and STAT92E promote Draper expression [80]. PI3K signaling is important in regulating basal Draper expression but not increased Draper expression in response to injury [81]. *Drosophila* activator protein 1 (dAP-1), composed of the c-Jun and c-Fos orthologs, Jun-related antigen (Jra) and Kayak (Kay), respectively (Table 1), are transcriptional regulators that appear to promote *Draper* expression [37]. dAP-1 regulation of Draper expression is most likely indirect, by increasing *STAT92E* transcription [77]. dAP-1 is the transcription factor at the end of the JNK cascade, which requires signalling via both Tak1 and Slipper mitogen-activated protein kinase kinase kinases (MAPKKKs), MKK4 and Basket (bsk), the ortholog of JNK (Table), to initiate the transcriptional response to axonal injury [37]. Rac1 is also able to indirectly up-regulate *Draper* expression in a STAT92E-dependent manner.

Activation of Draper signaling is dependent on the intracellular adaptor dCed-6 binding the immunoreceptor tyrosine-based activation motif (ITAM), phosphorylation by Src42a and subsequent binding of Shark, a non-receptor tyrosine kinase of the Syk family [77,81]. Binding of dCed-6 to Draper is required for engulfment of axonal debris [77,81]. In response to ORN axotomy and adult VNC crush, Matrix metalloprotease 1 (MMP1), which facilitates infiltration and clearance of axonal debris is also up-regulated via Draper-dependent signaling in EG [30,82]. TNF receptor associated factor 4 (TRAF4) has been identified to bind Draper via the NPXY motif, independent of Shark. TRAF4 relays the signal from the activated Draper receptor to the JNK cascade via Misshapen, a MAPKKK [77].

The protein phosphatase 4 (PP4) complex is required for appropriate recruitment of EG to injured axons and infiltration of the damaged site. PP4 activates Rac1, Rho GTPase, actin cytoskeletal remodelling, membrane extension and engulfment, via guanine nucleotide exchange factor Sos [83].

*Draper* transcription produces three isoforms, Draper-I, -II and -III. Draper-I is the isoform associated with the phagocytic capacity of EG. Draper-I activation creates a positive feedback loop, leading to increased Draper-I expression. This process is antagonized by Draper-II, whose intracellular ITAM domain differs from Draper-I. Interestingly, Draper-II is only expressed in adult flies, suggesting EG engulfment may be regulated differently between larval and adult brains. Draper-II contains an immunoreceptor tyrosine-based inhibitory motif (ITIM) that is selectively bound by the tyrosine phosphatase Corkscrew (Csw). Csw dephosphorylates Draper-II, resulting in Draper-I signaling inactivation. The signaling pathway beyond Csw is unknown [84]. In mammalian microglia, the interplay between ITAM and ITIM signaling molecules is also associated with positive and negative regulation of phagocytosis, respectively [85].

In addition to debris clearance, EG also appear to induce signaling in surrounding undamaged axons, potentially hindering regenerative attempt. Following axotomy in the L1 wing vein, EG suppress axonal transport in undamaged neurons proximal to the lesion by a process that is dependent on JNK signalling, MMP1 expression and PI3K/Raptor signaling [86].

In conclusion, Draper expression, activation and localization requires multifactorial input which safeguards against an accidental switch on of phagocytosis.

### Repair and regeneration: glial proliferation and differentiation

Neurogenesis and gliogenesis in the adult fly brain are controversial topics. The neural stem cells (also known as neuroblasts) that are present during embryonic, larval and pupal development are lost through apoptosis or exit from the cell cycle before eclosion of adult flies. In healthy adult brains proliferation has been detected by a variety of techniques [31,87,88], but no adult neural stem cell population has been identified [89]. However, it is generally accepted that proliferation in the adult brain is triggered in response to injury (Figure 2). This occurs not only in glial cells, producing new neurons and glia [30,87,90] but also in cells negative for glial and neuronal markers but positive for Deadpan (Dpn), a transcription factor and stemness marker (Table 1) that becomes nuclear localized upon injury [31,34]. These adult cells, which behave similarly to quiescent NSCs, have been detected in the optic lobe and the central brain. Proliferation signals activate *Myc* expression in optic lobe NSCs and ectopic *Myc* expression in these NCSs can stimulate proliferation in the undamaged optic lobe [31].

In the undamaged larval VNC, limited glial proliferation occurs [91]. This proliferation increases in response to injury. Injuries activate a gene network that controls the proliferation of ALG. The activation depends on *Notch*, *Pros* and *kon*, and the network is largely conserved in the mammalian spinal cord [22,92]. The proliferation of ALG is controlled by a balance of Notch and Pros expression, which promote and limit proliferation, respectively, and requires Cyclin E to initiate division. The importance of the Notch/ Pros balance is highlighted when the Notch intracellular domain (Notch^ICD^) is overexpressed. Notch^ICD^ acts as a dominant activator of Notch signaling resulting in a rise in glial proliferation and increased VNC length of uninjured VNCs in wandering stage larvae. VNC length further increases when in *pros* mutants either Notch^ICD^ or Dorsal/ NfkappaB is overexpressed [91].

When the larval VNC is stabbed, the proinflammatory tumor necrosis factor (TNF) Egr is produced and binds Wengen, a TNF receptor. Activation of the adapter dTRAF2 and subsequent downstream signaling leads to nuclear translocation of Dorsal, the fly ortholog of NFkappaB (Table 1). Dorsal/ NfkappaB aids Notch signaling to induce proliferation by up-regulation of Kon expression, which is only expressed at very low levels within the undamaged VNC. Kon is a central regulator of the glial proliferative response, shown to promote proliferation and repair when overexpressed in the larvae and limiting repair when knocked down. Kon contains an intracellular PDZ motif. The PDZ motif is cleaved by alpha- and gamma-secretase, producing four isoforms including an intracellular domain which may regulate gene expression [93]. Kon exists in two negative feedback loops [28]. It inhibits Notch signaling, limiting Notch-dependent proliferation. Kon also promotes Pros expression, a transcription factor that inhibits proliferation driving glial differentiation and axonal enwrapment. Pros activates Dacapo (the ortholog of p21/p27, Table 1) expression instructing the glial cell to enter into the resting G0 phase of the cell cycle. Finally, Pros activates *Dorsal* and *Notch* expression to restore a balance between proliferation and differentiation, returning glial cells to quiescence but maintaining the ability to respond to injury [22,91].

Recently, *Islets antigen-2 (Ia-2)*, which is exclusively expressed in neurons, has been shown to genetically interact with *kon*, exclusively expressed in glia [90]. This glial-neuronal communication has been implicated in glio- and neurogenesis response to CNS injury (Figure 2), but the mode of interaction is currently unknown. In response to VNC crush injury, both kon and Ia-2 expression increase, *Drosophila* insulin-like peptide 6 (Dilp-6) is secreted from neurons and glia and received by the Insulin receptor (InR) on glia. Activation of Insulin signaling stimulates a positive feedback loop in glia, amplifying Dilp-6 secretion and promoting proliferation, which is dependent on Kon and Ia-2. Dilp-6 induces proliferation of all glia but only induces *Dpn* expression in NG2-like glia. Dpn expression in NG2-like glia confers neural stem cell-like capacity to these glial cells. The newly created glial-derived NSCs can divide to produce a very limited number of glia and neurones [90].

**Figure 3:**
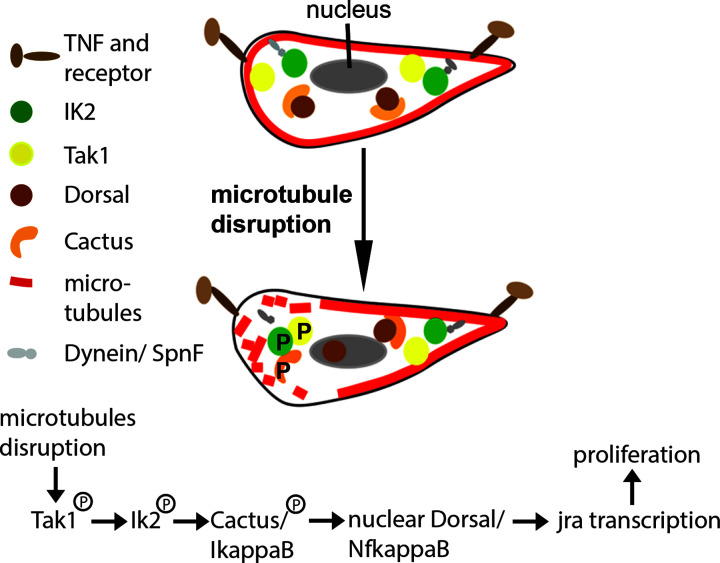
Traumatic Injury in the embryonic CNS results in cell proliferation Injury in the developing *Drosophila* CNS at the midline, the tissue analogous to the vertebrate floor plate, disrupts microtubule integrity. The kinases Tak1 and Ik2 are released from the microtubules and activated by phosphorylation. Ik2 phosphorylates Cactus, the Ikappa B ortholog of *Drosophila*, which in turn targets Cactus for degradation and releases Dorsal, the NfkappaB *Drosophila* ortholog. Dorsal enters the nucleus and triggers jra/ jun transcription terminating in proliferation; Ik2, IkappB kinase, Jra, Jun-related antigen; Tak1, TGFb-activated kinase 1.

TNF signalling is also crucial for damage repair in the embryonic *Drosophila* CNS (Figure 3). Ablation of midline cells, the neural cells analogous to the vertebrate floor plate, triggers additional divisions of adjacent undifferentiated midline sibling cells, but not of differentiated siblings. These damage-induced divisions are activated by disruption of the microtubule cytoskeleton but not via disruption of actin or cadherin adhesion. In undamaged embryos, additional divisions can be triggered by disrupting microtubules via depletion of GTPase mitochondrial Rho (Miro), overexpression of α-tubulin, injection of the microtubule depolymerizer Vinblastin or expression of human Tau [26,94]. All four manipulations interfere with microtubule integrity. Damage-induced divisions of midline cells are dependent on Jra [26] but do not require Kay, hence they are dAP-1 independent [94]. Upstream of Jra is TNF signaling, dependent on Egr, TGF-β activated kinase 1 (Tak1) and IkappaB kinase (Ik2). Microtubule disruption activates phosphorylation of Tak1, and subsequently Ik2. Cactus (IkappaB) becomes phosphorylated allowing Dorsal (NfkappaB) to translocate into the nucleus leading to *Jra* expression and activating Jra-controlled transcription. Damage induced proliferation, due to microtubule cytoskeletal disruption remains unexplored in mammalian injury. Yet, in Alzheimer’s disease (AD) brains, where diseased Tau disrupts the microtubule cytoskeleton, Tak1 and the ortholog of *Drosophila* IK2 (Table 1), Tank binding kinase 1 (Tbk1), are seen phosphorylated and colocalise with diseased Tau. NFkappaB is up-regulated and translocates to the nucleus, coinciding with abnormal mitosis in inflammatory cells and neurons [94]. It therefore seems possible that as in the ventral midline, microtubule disruption in AD brains activates mitosis in mature neurons using the same signaling network.

## Conclusion

Understanding the repair processes that occur in response to CNS injury is critical to developing therapeutic strategies for traumatic brain injuries. The absence of an adaptive immune system, oligodendrocytes and a glial scar may not favor the use of *Drosophila* as a model to unravel CNS repair mechanisms after injury. Yet, its use has served us well to shed light on the genetic control of Wallerian degeneration, the rapid clearance of cell debris, axon regeneration and cell proliferation and differentiation to replace damaged cells. In mammals, proliferation of adult NSCs and glia has been demonstrated to produce new neurons and glia. The extent of the proliferative response is often limited, and it is unclear how newborn neurons functionally integrate. It is pivotal to understand how newly generated cells can be manipulated to differentiate into specific cell types and how to achieve correct axonal and synaptic targeting. The recent discovery of NG2-like glia in the *Drosophila* larval CNS which gain proliferative capacity after CNS damage will allow to follow neuronal regeneration and axonal targeting in a living organism. Using the model of *Drosophila* c4da axons is enabling the identification of the basic mechanisms allowing severed axons to regrow in the periphery but preventing axonal regrowth in the CNS. The surprising role of microtubule integrity in the activation of cell division at the *Drosophila* midline may lead to better understanding of abnormal neuronal divisions caused by neurodegeneration. It is encouraging that repair and regenerative pathways identified in *Drosophila* have been conserved in mammals (Table 1). The genetic power of *Drosophila* will continue to be important in understanding CNS damage and repair and provide valuable insights into the challenges faced by regenerative medicine.

## Data Availability

The submitted article is a review based on previously published works. No new data have been produced in the course of writing the review.

## Abbreviations

ALG: astrocyte-like glia
Axed: Axundead
C4da: Class 4 dendritic arborisation
CNS: central nervous system
CPE: cytoplasmic polyadenylation element
Csw: corkscrew
dAP-1: *Drosophila* activator protein 1
Dilp-6: *Drosophila* insulin-like peptide 6
DLK1: dileucine zipper kinase 1
DRG: dorsal root ganglion
Dscam1: Down syndrome cell adhesion molecule 1
EG: ensheathing glia
Egr: Eiger
Faf: Fat-facets
GFAP: glial fibrillary acidic protein
Grnd: Grindelwald
Hiw: Highwire
Ia-2: Islets antigen-2
Ik2: IkappaB kinase
InR: insulin receptor
ITAM: immunoreceptor tyrosine-based activation motif
ITIM: immunoreceptor tyrosine-based inhibitory motif
JNK: c-Jun n-terminal kinase
Jra: Jun-related antigen
Kay: Kayak
Kon: Kon-tiki
MMP1: matrix metalloprotease 1
MtN: metathoracic neuromere
Mycbp2: Myc-binding protein 2
NG2: neural glia antigen 2
NMNAT: nicotinamide-nucleotide adenylyltransferase 1
NSC: neural stem cell
ORN: olfactory receptor neuron
PKA: protein kinase A
PNS: peripheral nervous system
PP4: protein phosphatase 4
Pros: Prospero
repo: reverse polarity
Rtca: RNA 3′-terminal phosphate cyclase
Rtcb: RNA 2′,3′-cyclic phosphate and 5′-OH ligase
Sarm: Sterile alpha/Armadillo/Toll-interleukin receptor homology domain protein
SGZ: subgranular zone
sLNv: small lateral neurons ventral
Src42: Sarcoma 42
STAT92E: signal-transducer and activator of transcription protein 92E
SVZ: subventricular zone
Tak1: TGF-β activated kinase 1
TBI: traumatic brain injury
Tbk1: Tank-binding kinase 1
TNF: tumor necrosis factor
TRAF4: TNF receptor associated factor 4
VNC: ventral nerve cord
WD: Wallerian degeneration
WldS: slow Wallerian degeneration
Wnd: Wallenda
wt: wild-type
Xbp1: X-box binding protein 1
